# Pyoderma gangrenosum developing in a patient treated with dupilumab

**DOI:** 10.1016/j.jdcr.2021.12.009

**Published:** 2021-12-17

**Authors:** Jessica Nasseh, Henry Abi Rached, Ioana Dumitru, Delphine Staumont-Sallé, Frédéric Dezoteux

**Affiliations:** a Service de Dermatologie, Centre Hospitalier Universitaire de Lille, Lille, France; b Service de Dermatologie, Centre Hospitalier Universitaire de Lille and INSERM U1189 - Laser Assisted Therapies and Immunotherapies for Oncology, Université de Lille, Lille, France; c Institut de Pathologie, Centre Hospitalier Universitaire de Lille, Lille, France; d Service de Dermatologie, Centre Hospitalier Universitaire de Lille and INSERM U1286 - Institute for Translational Research in Inflammation, Université de Lille, Lille, France

**Keywords:** atopic dermatitis, drug reaction, dupilumab, pyoderma gangrenosum, AD, atopic dermatitis, IL, interleukin, PG, pyoderma gangrenosum, Th, T helper cell

## Introduction

Dupilumab is a fully human monoclonal antibody targeting the interleukin 4 (IL-4) receptor α subunit, thereby blocking IL-4 and IL-13 signaling, which play an important role in the T helper cell 2 (Th2)-mediated inflammatory reaction found in atopic dermatitis (AD). Dupilumab has a good safety profile, with conjunctivitis being the main side effect. Few cases of psoriasis, a result of Th1- and Th17-mediated inflammation, were described following treatment with dupilumab in patients with AD. Herein, we present a case of pyoderma gangrenosum (PG) that developed in an adult with AD who had been treated with dupilumab.

## Case report

A 33-year-old man with a history of Hashimoto disease, seasonal allergies, asthma, and severe AD since childhood was started on dupilumab therapy after failure of optimal topical treatment and intolerance to methotrexate. Five months before presentation, he was diagnosed with Crohn's disease, which was well controlled with budesonide and 5-aminosalicylic acid.

**Fig 1:**
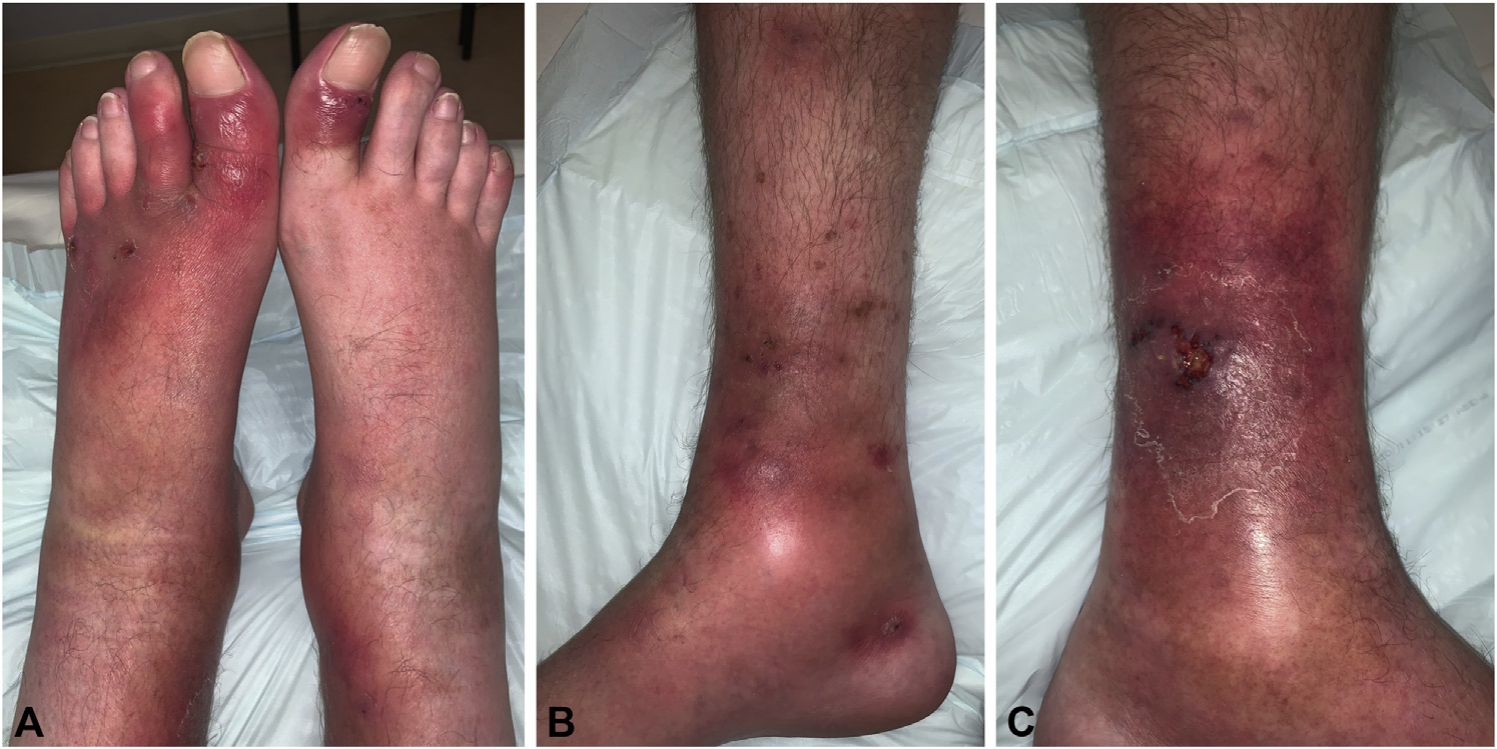
Clinical presentation. **A**, Dactylitis and bilateral arthritis. **B**, Tender nodules and a pus-filled vesicle on the inner side of the ankle. **C**, Ulceration on the lower portion of the left leg with an edematous, violaceous border.

Two days after his loading dose of dupilumab, he was hospitalized for rapidly progressing lower leg edema, painful ulceration, and worsening skin lesions. Physical examination found extensive eczematous plaques on the face, neck, lower portion of the back, and the flexor surfaces of the elbows and knees. He also developed bilateral ankle arthritis and dactylitis of both big toes and the left second toe (Fig 1, *A*), a lower leg ulcer, and numerous tender red to purple nodules, some breaking down to form small ulcers (Fig 1, *B*). The ulcer base was purulent, the borders were undermined and violaceous, and the surrounding skin was erythematous with infiltration and edema (Fig 1, *C*).

On presentation, the patient was afebrile and his vital signs were stable. He had no abdominal pain nor diarrhea. Complete blood count and comprehensive metabolic panel, liver profile, and hemostasis tests were normal. The C-reactive protein level was elevated (63 mg/L; normal range <3 mg/L). Serologic tests for hepatitis B and C, HIV, and syphilis were negative, as were tests for rheumatoid factor and antinuclear antibodies. Arterial and venous Doppler studies revealed no abnormalities.

**Fig 2:**
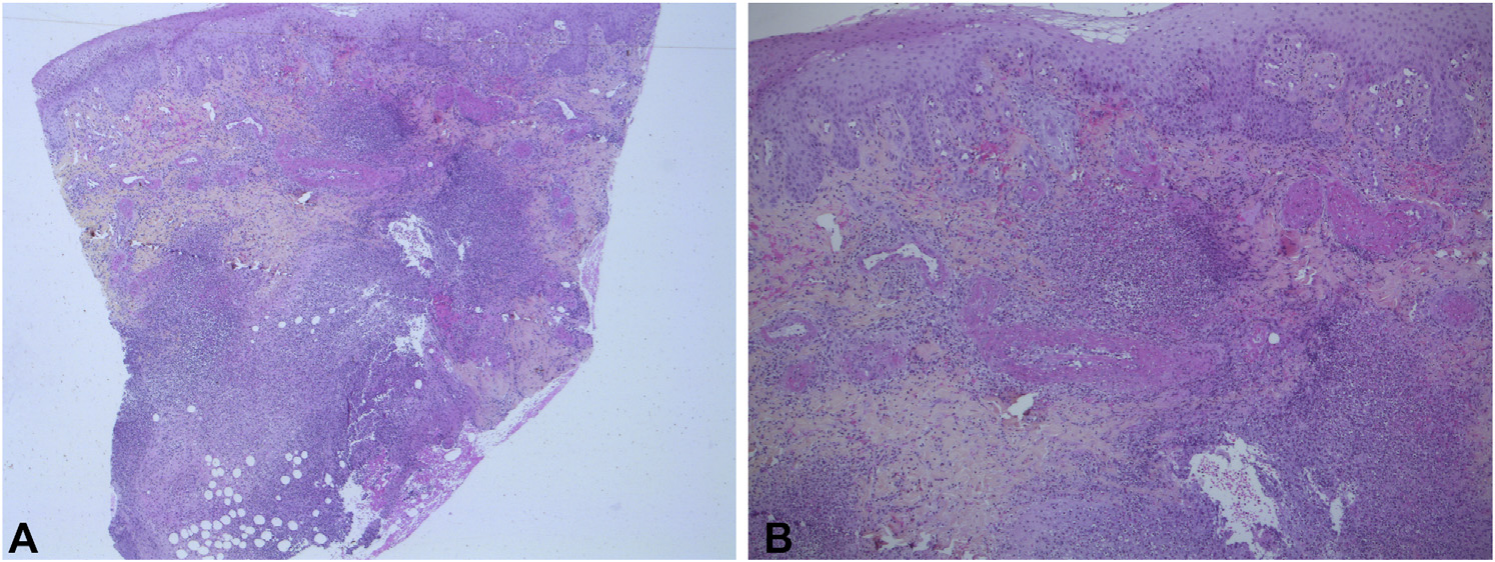
Histopathologic findings. **A**, Acanthosis and papillomatosis. **B**, Dense inflammatory infiltrate within the dermis, predominantly of neutrophils. (**A** and **B**, Hematoxylin-eosin stain; original magnifications: **A**, ×5; **B**, ×20.)

Skin biopsy of a pus-filled vesicle revealed a hyperplastic ulcerated epidermis (Fig 2, *A*), hemorrhage and edema in the papillary dermis, and an abundant and dense inflammatory infiltrate within the dermis and the hypodermis, predominantly of neutrophils and eosinophils (Fig 2, *B*). Periodic acid–Schiff stain of the specimen was negative. Tissue cultures for bacteria, mycobacteria, and fungi yielded negative results. The findings were compatible with PG. A biopsy specimen from a livedoid lesion was negative for vascular immune complex deposition by direct immunofluorescence.

We diagnosed PG and paradoxical AD flare triggered after treatment with dupilumab, in the context of known Crohn's disease. Dupilumab was discontinued, the lesions improved with treatment with topical steroids and wound care, and the ulcer was completely healed at the 3-month follow up.

## Discussion

This is an original case of PG developing in a patient treated with dupilumab. PG is a rare neutrophilic dermatosis frequently related to chronic inflammatory bowel disease. Its pathogenesis is complex, multifactorial, and not completely understood, but it appears to be an immune-mediated injury similar to inflammatory bowel disease.

Current trends of research have emphasized an imbalance between proinflammatory Th17 cells and regulatory T cells in PG lesions, resulting in overactivity of T cells and increased levels of IL-17 produced by Th17, thus creating a proinflammatory environment and attracting neutrophils to the dermis.^1^^,^^2^ In 2016, Quaglino et al^3^ were the first to analyze cytokine and chemokine expression in the peripheral blood of patients with PG and hypothesized a Th1/Th17-mediated inflammatory pattern with down-regulation of Th2.

Some cases of PG triggered by medications other than dupilumab have been reported. Multiple pathogenetic mechanisms have been proposed, affecting in particular chemokine production and release, recruitment of inflammatory cells and neutrophil migration, enhancement of effector T-cell function, reduction of regulatory T-cell function, and increase in IL-17 production, all of which reinforce the hypothesis of a Th1/Th17-mediated immunity.^4^ Moreover, IL-17 is also shown to play an important role in inflammatory bowel disease and other skin diseases that involve neutrophil recruitment, including psoriasis.

Previous case reports of dupilumab-induced psoriasis in patients with AD suggest a possible shift from a Th2- to a Th1/Th17-mediated inflammatory response.^5^^,^^6^ IL-4, which plays a pivotal role in the development of Th2 responses, has been suggested to be a key regulator of Th17 activity in AD skin.^7^ Thus, the proposed hypothesis is that blockade of Th2 cytokines by dupilumab could impair this regulatory mechanism, inducing activation of a Th1/Th17 pathway and psoriatic lesions as a result.^8^ By analogy, we hypothesize that blockade of Th2-mediated inflammation by dupilumab and a resultant shift toward a Th1/Th17 phenotype was a pathogenic factor in the development of PG in our patient, although we acknowledge that PG might have developed independently of treatment with dupilumab in the setting of Crohn's disease.

To our knowledge, new presentation or exacerbation of PG has not been reported in the setting of dupilumab therapy, suggesting that the proposed mechanism of immune dysregulation or shift may be clinically relevant only in genetically predisposed individuals. Although the development of PG in our patient may only be coincidental, we suspect a causative role of dupilumab. Further observations and, ideally, T-cell cytokine profiling, are needed to determine whether dupilumab is truly associated with an increased risk of developing a neutrophilic disease and to elucidate the mechanisms behind this potential adverse event in patients with concurrent Crohn's disease or a predilection to develop PG.

## Conflicts of interest

Dr Staumont-Sallé is an investigator for AbbVie, Amgen/Celgène, Astra-Zeneca, Boehringer Ingelheim, Galderma, Eli Lilly, Leo Pharma, Novartis, and Sanofi-Regeneron; a consultant for AbbVie, Astra-Zeneca, Eli Lilly, Leo Pharma, Janssen, Novartis, Sanofi, and Pfizer; and a speaker for AbbVie, Eli Lilly, Janssen, Novartis, Pfizer, Sanofi, and UCB. Dr Dezoteux is an investigator for AbbVie, Amgen/Celgène, Astra-Zeneca, Boehringer Ingelheim, Galderma, Leo Pharma, Novartis, Sanofi-Regeneron. and Lilly; a consultant for Leo Pharma, Novartis, Sanofi, and Lilly; and a speaker for Leo Pharma, AbbVie, and Lilly. Drs Nasseh, Abi Rached, and Dumitru have no conflicts of interest to declare.
